# Prometheus Revisited: From Liver to Heart

**DOI:** 10.1111/liv.70722

**Published:** 2026-06-13

**Authors:** Michele Augusto Riva

**Affiliations:** ^1^ School of Medicine and Surgery University of Milano‐Bicocca Monza Italy; ^2^ Department of Occupational Health Fondazione IRCCS San Gerardo dei Tintori Monza Italy

**Keywords:** fine arts, hepatocentrism, liver, mythology, Prometheus

In Greek mythology, Prometheus was famously condemned by Zeus to remain chained to a rock while an eagle eternally devoured his liver, which regenerated every night. Because the liver was traditionally regarded as the seat of life and blood production in Greco‐Roman medicine, the myth became one of the most enduring symbolic representations of ancient hepatocentric doctrines [1, 2].

An intriguing reinterpretation of this myth may be observed in *Prometheus* (1628) by the Flemish painter Theodoor Rombouts (1597–1637). In this work, the eagle appears to attack the upper thoracic region of Prometheus, shifting the focus away from the liver and towards the chest and heart (Figure 1A). This detail differs from the traditional iconography of Prometheus, in which the liver is explicitly targeted. A notable example is *Prometheus Bound*, painted only a few years earlier (1611) by the fellow Flemish painter Peter Paul Rubens (1577–1640), where the eagle violently tears the right upper abdomen and visibly grasps a fragment of Prometheus' liver in its beak (Figure 1B).

**FIGURE 1:**
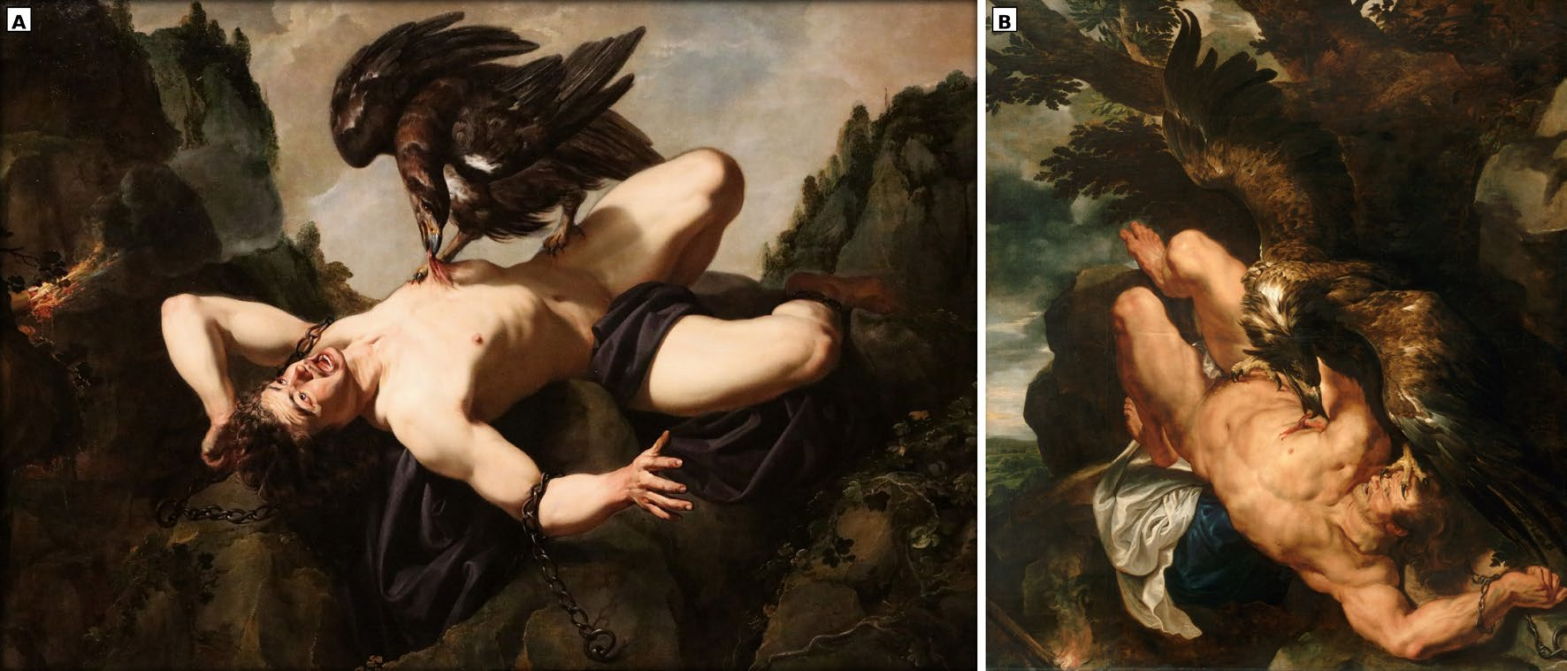
(A) *Prometheus* (1628) by Theodoor Rombouts, Royal Museums of Fine Arts of Belgium, Brussels, Belgium. (B) *Prometheus Bound* (1611) by Peter Paul Rubens, Philadelphia Museum of Art, Philadelphia, USA. Both images were obtained from Wikimedia Commons under Creative Commons licences.

The comparison between these two culturally related Flemish works, produced within less than two decades, may reflect the profound medical transition occurring during the seventeenth century, when cardiocentric theories progressively replaced ancient hepatocentrism. Remarkably, Rombouts completed his painting in the same year in which William Harvey (1578–1657) published his renowned treatise that revolutionized the understanding of blood circulation and reinforced the symbolic centrality of the heart [3, 4, 5]. In this perspective, Rombouts' *Prometheu*s may represent an early artistic testimony of the shift from liver‐centred to heart‐centred physiology.

## Author Contributions

The author takes full responsibility for this article.

## Conflicts of Interest

The author declares no conflicts of interest.

## Data Availability

The author has nothing to report.
